# Supplementary material for: Common hematological parameters reference intervals for apparently healthy pregnant and non-pregnant women of South Wollo Zone, Amhara Regional State, Northeast Ethiopia
Source: PLoS One. 2022 Jul 15;17(7):e0270685. doi: 10.1371/journal.pone.0270685 (PMC9286272; doi:10.1371/journal.pone.0270685)
Supplement: Attachment — Submitted filename: Response to Reviewer.docx [file pone.0270685.s001.docx]

**Response to Reviewer and academic editor**

**To Academic Editor**

**Responses**: First of all, we would like to thank you for your reminder to consider the journal formatting requirement. Therefore, we made a correction on some of the formatting issues. In addition, we have deleted two duplicate references and added a new reference as per the academic reviewer's recommendation, and a correction has been made to the reference list, which is indicated in the document.

**To Reviewer:** We would like to thank you very much for your pertinent comments that praised the paper quality.

**Comment 1**: Some large reference studies on WBC and platelets are not referenced, and you may benefit from their inclusion (references below). Your reference limits differ from these in places - can you comment on why?

**Response:** Thank you. As per your recommendation, we have used the two papers and the possible explanation for the variation of the reference limit of WBC and platelet with our study is postulated to occur due to factors such as nutritional status, genetics, dietary patterns, ethnic origin, and environmental exposure to endemic pathogens.

**Comment 2:** Overall, the text is verbose and should be edited for brevity before publication (particularly in the discussion and conclusion)

**Response:** Well addressed accordingly, thanks (We tried to avoid verbose text in the document, particularly in the discussion and conclusion.**)**
